# Possibility of mpox viral transmission and control from high-risk to the general population: a modeling study

**DOI:** 10.1186/s12879-023-08083-5

**Published:** 2023-02-24

**Authors:** Shiting Yang, Xiaohao Guo, Zeyu Zhao, Buasiyamu Abudunaibi, Yunkang Zhao, Jia Rui, Yao Wang, Wentao Song, Hongjie Wei, Tianmu Chen

**Affiliations:** State Key Laboratory of Molecular Vaccinology and Molecular Diagnostics, School of Public Health, Xiamen University, 4221-117 South Xiang’an Road, Xiang’an District, Xiamen City, Fujian Province People’s Republic of China

**Keywords:** Mpox virus, SEIR model, Transmissibility, Intervention

## Abstract

**Background:**

Mpox is an emerging infectious disease that is now a global hazard. The strategies for preventing and controlling mpox should be further investigated in terms of transmission characteristics, infection risk among different populations, and ideal therapeutic approaches.

**Methods:**

A multi-group dynamic model was used to quantify the transmissibility of mpox. We further analyzed the transmission risk from men who have sex with men (MSM) to non-MSM and evaluated the effects of three intervention measures, including community-based prevention, early detection, and vaccination.

**Results:**

The median value of effective reproduction number (*R*_*eff*_) and probability of contact via a single contact (*q*) among MSM worldwide is 3.11 (interquartile range [IQR]: 2.82–5.57) and 2.15% (IQR: 1.95–3.84%). We found that the cumulative incidence rate of non-MSM is much lower than that of MSM (< 1/2048) when the possibility of infection (including the percentage of high-risk behaviors of contact degree [*C*] and *q*) was lowered to less than 1 in 100,000. When comparing the three intervention measures, if we want to control the cumulative incidence rate to 5.96 × 10^–8^ we need to increase the vaccine coverage to 81% or reduce the transmission rate factor (*Cq*) to 70% or shorten the transmission period to 74%.

**Conclusions:**

Mpox has high transmissibility in MSM, which required minimize the risk of infection and exposure to high-risk populations. Community prevention and control is the top priority of interventions to contain the spread of mpox.

**Supplementary Information:**

The online version contains supplementary material available at 10.1186/s12879-023-08083-5.

## Introduction

Mpox, known as a rare zoonotic disease caused by the mpox virus (MPXV), is now a potential threat worldwide [1]. Since it was first reported in central Africa in 1970, the virus has affected some undeveloped areas in the continent, especially the Democratic Republic of the Congo (Zaire) [2]. But in 2003, there was an outbreak in the United States, where the virus was first detected besides Africa [3]. From then on, several countries like Singapore [4] and the United Kingdom have reported confirmed cases of mpox [5]. However, an unusual outbreak of mpox occurred in May, 2022 [6]. By June 6, 2022, a total of 6106 confirmed cases and 51 suspected cases of mpox have been identified in more than thirty non-mpox endemic countries, and the number of cases is growing constantly [1]. According to World Health Organization (WHO), the case fatality rate of mpox is approximately 3–6%, which makes mpox a disease with a high mortality rate among infectious diseases. After the vaccination against smallpox ceased in 1980, majority of the new born population does not have immunity against smallpox or mpox virus and are susceptible to mpox [7], worse, there is no specific medical treatment developed for mpox for now. Every nation's healthcare system might be threatened by mpox. Therefore, the trend of mpox outbreak should be predicted and some effective preventive and control measures should be taken.

According to quantitative studies about mpox, a research team found that the disease has been given little attention for the past few decades, which only accounted for 5.6% of pox virus research [8], and this situation results in lacking knowledge of its transmission mechanism until this year’s outbreak. In the twentieth century, humans were mostly infected with the mpox virus via contacting or consuming infected animals, and interpersonal transmission was relatively rare [9, 10]. However, the outbreak since May 2022 differs from previous transmission pathways, in which the mpox virus has caused a larger scale of interpersonal transmission, and most reported cases have been concentrated in the group of men who have sex with men (MSM), which is associated with the ratio of exposure risk [11]. Although human-to-human transmission has become a topic of interest in recent years, studies of infectious disease transmission at the macro level are still not thorough enough, and so are the mathematical models to study the mechanisms of mpox transmission. Existing mathematical modeling studies related to mpox are limited to examining the ability of the mpox virus to spread throughout all populations and evaluating the effects of interventions that only consider the effects of a single vaccine effect or isolation measures [12–15]. These models do not adequately consider vaccine efficacy and coverage, nor did they include population heterogeneity. The widespread disease with human-to-human transmissibility is sounding alarms for the world, and WHO has therefore prioritized several research fields of transmission dynamics, containment, and the response of mpox, and suggests more attention to 'at-risk' groups [16]. Based on these research priorities, we found there are no studies quantifying the transmissibility of mpox in high-risk populations or evaluating the effectiveness of key interventions targeting this high-risk population for the time being.

In the study, we developed a multi-group model according to the natural history of mpox to provide a reasonable reference for the transmission and prevention of mpox in humans. This model allowed us to quantify the transmissibility, predict its outbreak trend, calculate the risk of transmission from MSM to non-MSM, and evaluate the efficacy of the intervention.

## Methods

### Study design

**Fig. 1:**
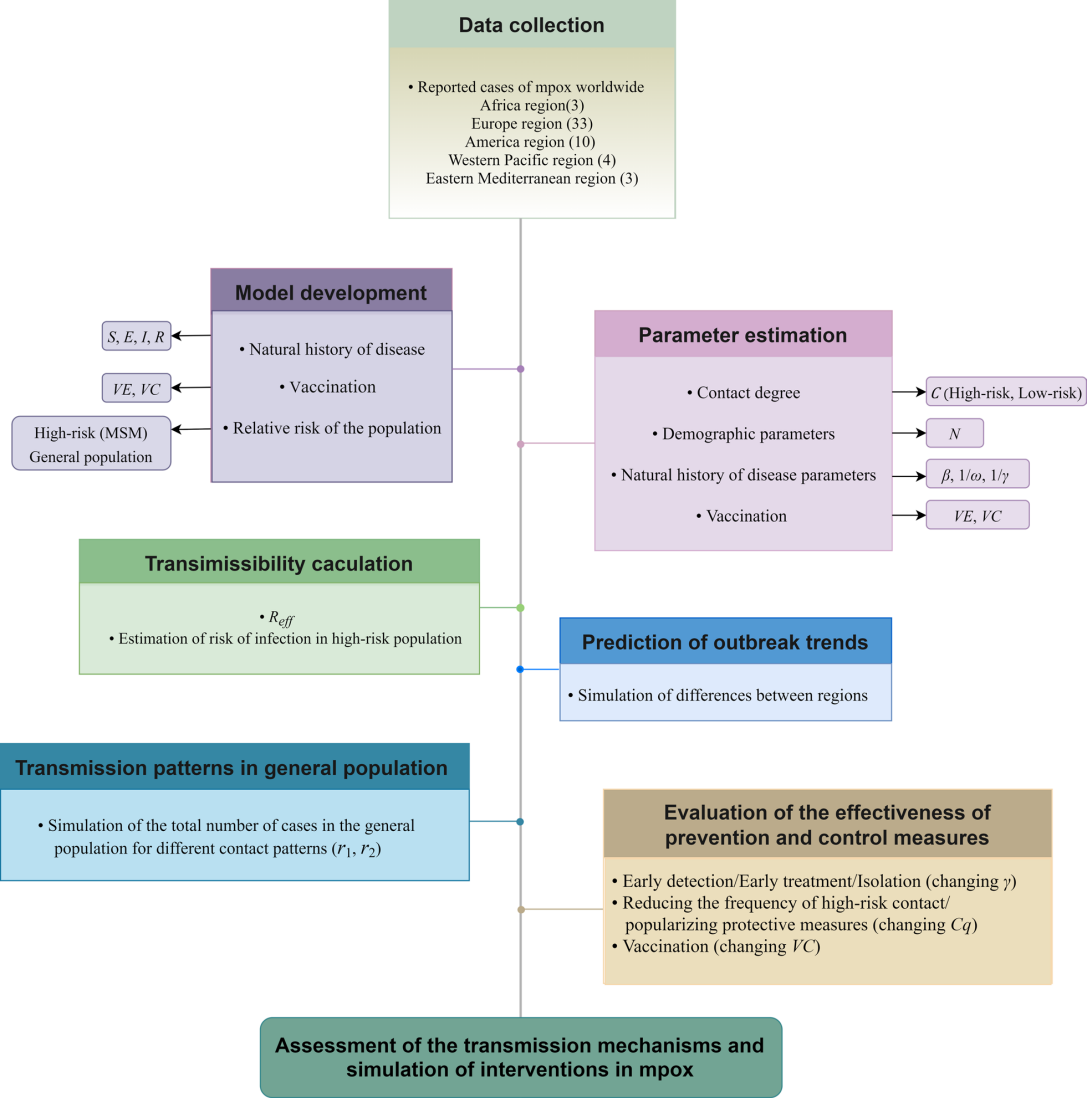
Study design for assessment of the possibility of mpox transmission and control from high-risk to the general population

In this study, a grouped model was first constructed for a high-risk population (MSM) based on the natural history of mpox, vaccination, and further sub-populations based on risk in different population groups (Fig. 1). Reported data for mpox were obtained globally and for five selected countries (the United Kingdom [UK], Spain, Portugal, Germany, and Italy) and their transmissibility was fitted based on the transmission dynamics models. The prediction of outbreak trends was further proceeded globally and in countries with a high number of cases. The intra- and inter-group transmission rate coefficients were parameterized using the corresponding contact matrices, which were decomposed into high- and low-risk exposures. The model is simulated with different scenarios of different ratios of high-risk and low-risk behaviors in contact degree and different relative risks. Finally, the effects of single or combined key interventions (early detection/early treatment/isolation, community prevention and control, increased vaccine coverage) were simulated by reducing the average period of transmission, reducing the local contact matrix, and scaling up the vaccine coverage parameters, respectively.

### Data collection

The reported cases of mpox were collected from a public database (Our world in data) [17]. Information on the database is detailed in Additional file 1. Our study collected epidemic curves from May 6, 2022, to July 1, 2022, with a simulated time step of 1 day.

### Model development

**Fig. 2:**
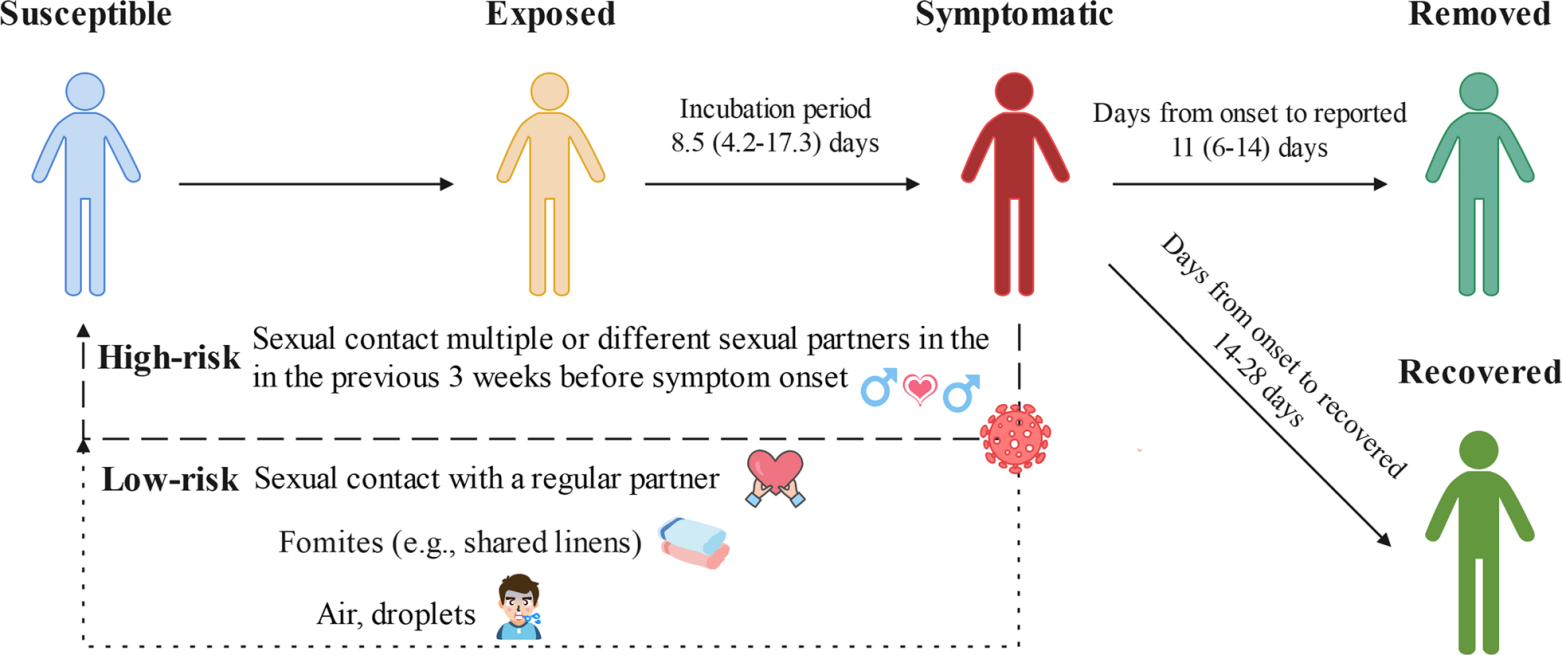
Clarifying diagram of the natural history of mpox. People with different colors indicate the different status of mpox, the solid line with arrows indicates the transition of each state, and the number above indicates the corresponding period and its range; the thick dashed line indicates the transmission route with a high risk of MPXV, and the thin dashed line represents the transmission route with low risk

The multi-group susceptible-exposed-infectious-removed (SEIR) model was developed based on the natural history and various human behavior (Fig. 2). The population was divided into $n$ subgroups, with each subgroup containing four compartments: susceptible population (${S}_{i}$), exposed population (${E}_{i}$), infectious population (${I}_{i}$), and removed population (${R}_{i}$). ${R}_{i}$ includes people in quarantine, and receiving treatment, recovering, as well as people who have died from the disease. Let ${N}_{i}$ denotes the population size of the *i*-th group. The model was based on the following assumptions:

- Transmission of MPXV occurs within and between the subgroups. Let subscript *i* and *j* denote two subgroups (can be the same group), then the rate of new infections in group *i* is formulated to be proportional to both ${S}_{i}$ and all ${I}_{j},\hspace{0.25em}\hspace{0.25em}j=1,2,\cdots ,n$. Therefore, transmission rate coefficients $\beta \in {\mathbb{R}}^{n\times n}$ is introduced as such proportion, with its entries ${\beta }_{ji}$ denote the transmission rate coefficient from infectious in group $j$ to susceptible in group $i$.
- The incubation period of mpox is defined as $1/\omega$. Infectious populations will be removed once they are reported, and the average infectious period of mpox was defined as $1/\gamma$.
- Demographic factors, including natural births, natural deaths, and population migration, are not considered in short-term outbreaks. The vertical transmission and the existence of asymptomatic cases are omitted for the lack of evidence.

**Fig. 3:**
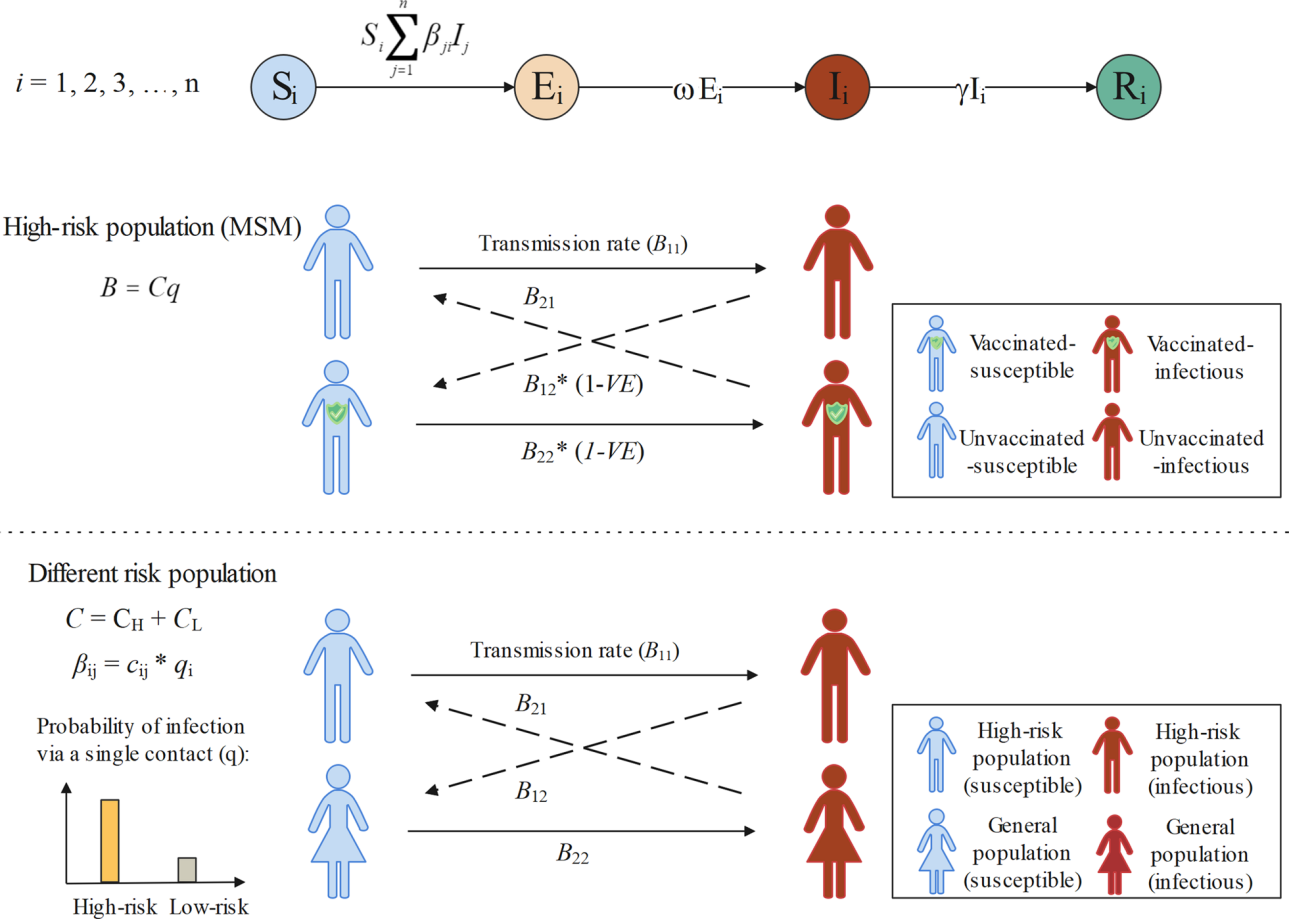
Flowchart of the SEIAR model. Subscript $i$, $j$ denote the $i$-th and the $j$-th group. Variables ${S}_{i}$, ${E}_{i}$, ${I}_{i}$, ${R}_{i}$ represent the susceptible, exposed, symptomatic infectious and recovered population in group $i$; ${N}_{i}={S}_{i}+{E}_{i}+{I}_{i}+{R}_{i}$ is the population size of group $i$; Parameters ${\beta }_{ji}$ is the transmission rate coefficient from group $j$ to group $i$; $\omega$ is the inverse of the average incubation period of symptomatic cases, it is used to quantify the removal rate of compartment $E$; $\gamma$ is the inverse of the average infectious period of symptomatic cases

Therefore, the basic SEIR model without intervention is shown in Fig. 3. The equations for the multi-group SEIR model are as follows:

$$\begin{array}{ll}\frac{d{S}_{i}}{dt}&=-{S}_{i}\sum_{j=1}^{n}{\beta }_{ji}{I}_{j}\\ \frac{d{E}_{i}}{dt}&={S}_{i}\sum_{j=1}^{n}{\beta }_{ji}{I}_{j}-\omega {E}_{i}\\ \frac{d{I}_{i}}{dt}&=\omega {E}_{i}-\gamma {I}_{i}\\ \frac{d{R}_{i}}{dt}&=\gamma {I}_{i}\\ {N}_{i}&={S}_{i}+{E}_{i}+{I}_{i}+{R}_{i}\end{array}$$

The transmission rate coefficient matrix $\beta$ is further decomposed as:

$$\begin{array}{c}\beta =Cq\end{array}\odot (1-VE)$$

where $C$ is the contact matrix with its $ij$-th entry representing the daily average number of contacts in group $j$ that one individual in group $i$ made; $q$ is the probability of infection via single contact; $\odot$ is the entry-wise product. The contact matrix is constructed globally, and $q$ is fitted worldwide and for all regions.

## Vaccination and population risk in the model

As the incidence is currently concentrated in the high-risk group (MSM), to estimate the transmission of mpox in them, we take the protective effect of the pre-1980 smallpox vaccine into account by dividing the population into un-vaccinated (group 1) and vaccinated (group 2) groups. In the model, considering that the vaccination could reduce infectivity or susceptibility, the transmission rate coefficient after vaccination is set to (1 − *x*)*β*, where *x* is defined as vaccine efficacy (*VE*).

Since the current outbreak of mpox cases mainly but not exclusively occurred in MSM [2], the number of female cases reported to date is low. Moreover, the behavioral contact pattern between lesbians differs from the invasive physical contact of MSM, and the likelihood of high-risk behavior is low. Therefore, the sex-specific model does not consider a subgroup of lesbians for the time being. It is worth noting that bisexual men in the MSM community can cause transmission through sex with females and that intra-family transmission is also a route of transmission. So the rest of the population is at risk of exposure to mpox. To quantify the potential risk of transmission in the entire population, we divided the population into three groups: MSM, non-MSM males, and females, further divided the contact matrix $C$ into high-risk contact ${C}_{1}$ and low-risk contact ${C}_{2}$:

$$\begin{array}{ll}{C}_{1}&={r}_{1}C\\ {C}_{2}& =\left(1-{r}_{1}\right)C\end{array}$$

where ${r}_{1}$ refers to the contact degree ratios of high-risk and low-risk behavior.

The relative risk ${r}_{2}$ of low-risk contact versus high-risk contact is introduced for low-risk contact ${C}_{2}$. Therefore, the weighted contact matrix ${C}_{3}$ is obtained:

$${C}_{3}={qC}_{1}+{{r}_{2}qC}_{2}={r}_{1}qC+{r}_{2}\left(1-{r}_{1}\right)qC=\left({r}_{1}+{r}_{2}-{r}_{1}{r}_{2}\right)qC$$

where *q* is the probability of infection via a single contact.

### Parameter estimation

The selection of parameters on natural history, contact behavior of different risk groups, vaccine efficacy and vaccine coverage are shown in Additional file 1.

### Intervention simulation

In this study, we simulated three scenarios (I, II, III) to assess the effect of key interventions in high-risk populations (MSM) (Table 1).

**Table 1:** Scenario parameter settings for simulation of intervention effects

Scenario	Measure	Varied parameter
I	Early detection/Early treatment/Isolation	1/*γ* = 1, 10/9, 10/8, …, 10
II	Community-based prevention and control (reducing the frequency of high-risk contact/ popularizing protective measures)	*Cq* = 0.1, 0.2, 0.3, …, 1
III	Increasing vaccination	*VC* = 0.1, 0.2, 0.3, …, 1

### Statistical analysis

The collected data were coded and sorted in Microsoft Excel 2013 (Microsoft Corp., USA), and curve fitting and simulation were performed by customized MATLAB 2021b (The MathWorks, Natick, MA).

## Results

### Epidemiological characteristics and model effectiveness

**Fig. 4:**
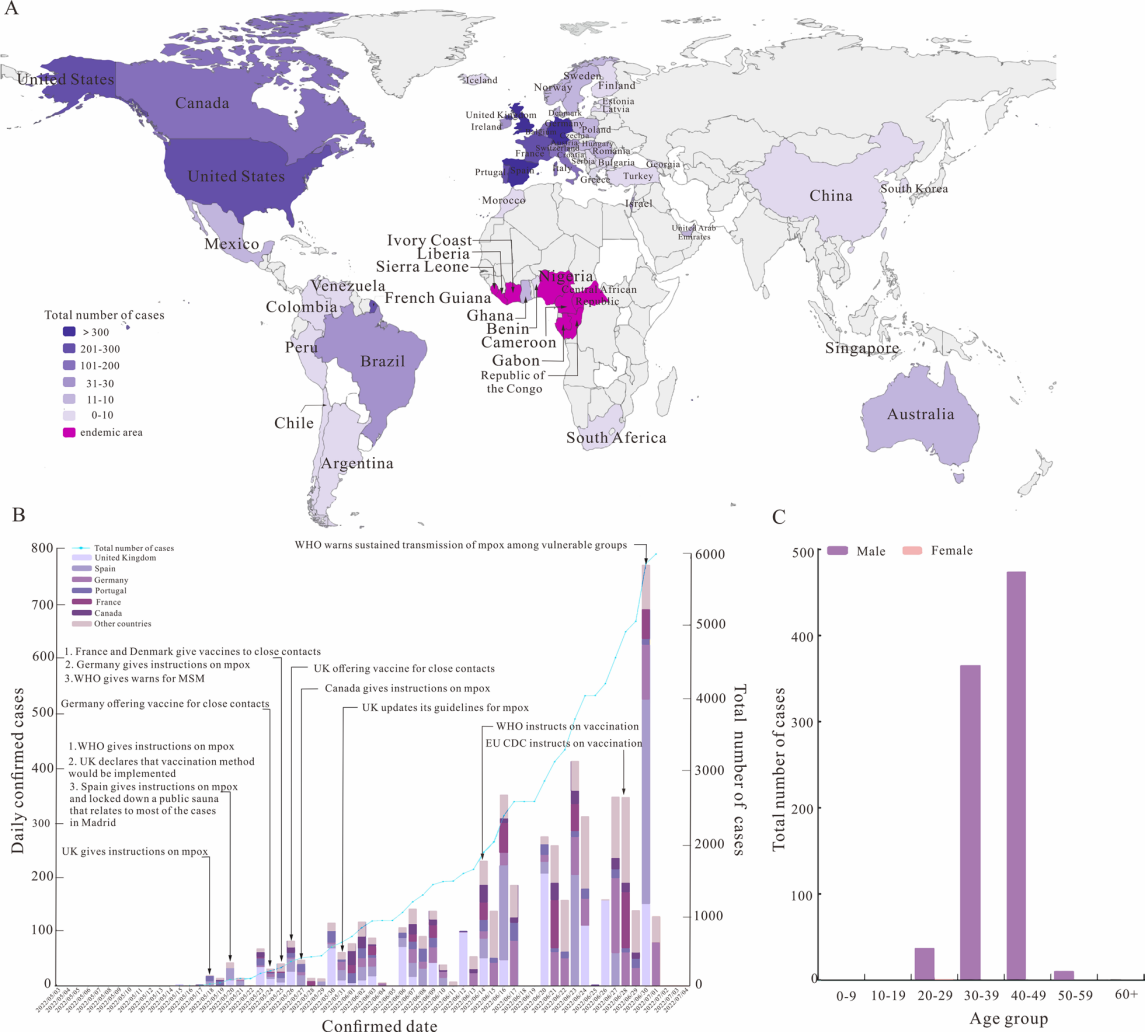
Epidemiological description of the global mpox outbreak in 2022. A: The spatial distribution of several mpox cases in different countries. B: Outbreak curve of confirmed mpox cases in global from 6 May, 2022 to 1 Jul, 2022. C: Gender distribution of mpox cases in global

A total of 6106 mpox confirmed cases were reported worldwide from 6 May, 2022 to 1 Jul, 2022. The number of daily confirmed cases is clearly on the rise (Fig. 4A). The outbreak of mpox mainly occurred in Europe and America, and most reported cases of mpox out of the endemic areas were in the United Kingdom (1235 cases), Germany (1054 cases) and Spain (1196 cases) (Fig. 4B). Among all confirmed cases of mpox reported during the outbreak, a large number of the cases were male, especially among MSM (Fig. 4C). We fitted the total number of cases globally and across the five countries with an MSM SEIR model and calculated the corresponding coefficient of determination (*R*^2^), showing that the model fit was good (Global: *R*^2^ = 0.985, *P* < 0.05; Spain: *R*^2^ = 0.974, *P* < 0.05; Portugal: *R*^2^ = 0.989, *P* < 0.05; Germany: *R*^2^ = 0.987, *P* < 0.05; Canada: *R*^2^ = 0.975, UK: *P* < 0.05; *R*^2^ = 0.973, *P* < 0.05) (Additional file 1: Fig. S1).

### Transmission in MSM

**Fig. 5:**
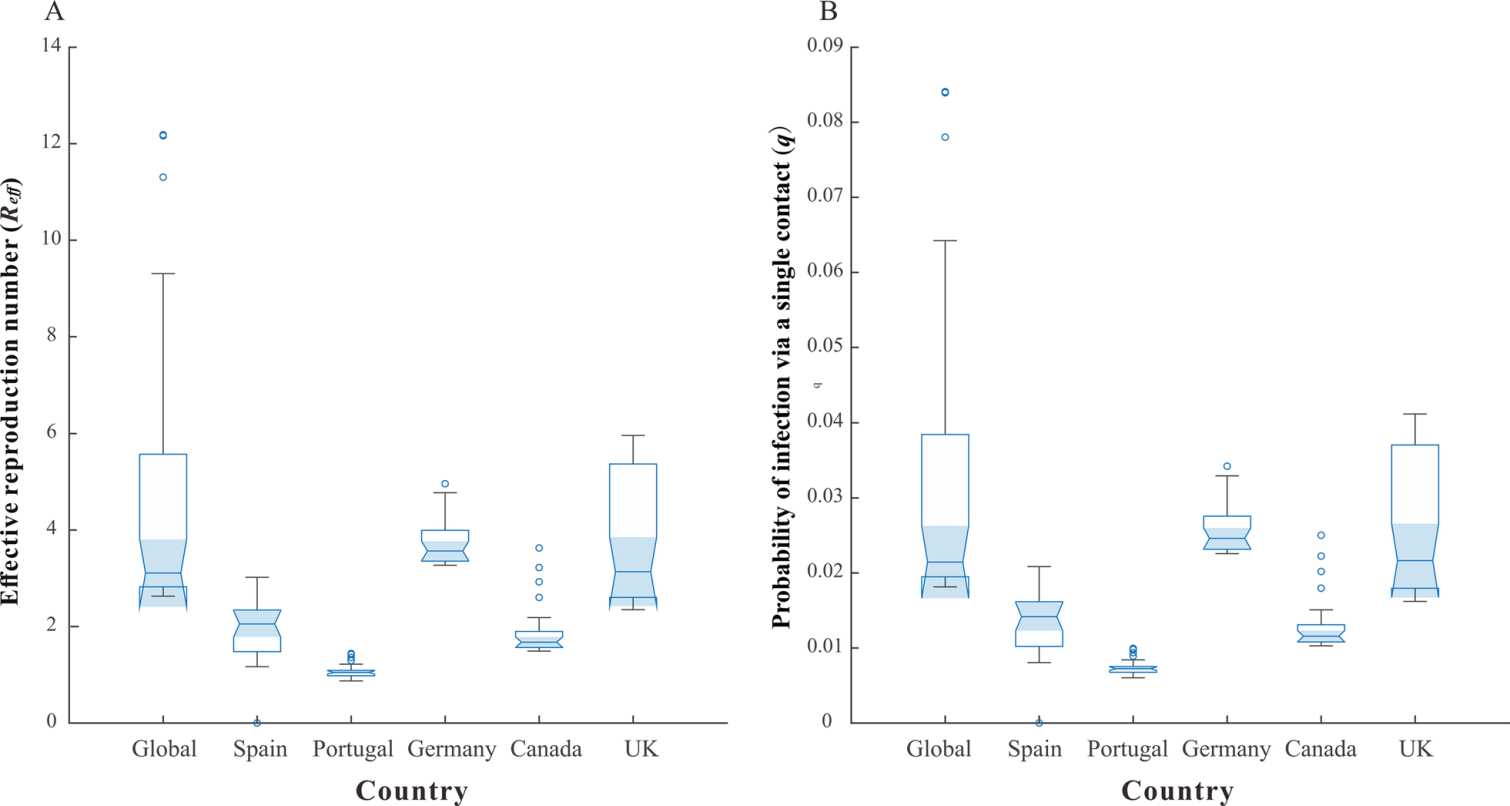
Transmissibility and risk of infection of mpox to the high-risk population in Global and five selected countries. **A** Fitting results of *R*_*eff*_; **B** Fitting results of the probability of infection via a single contact (*q*)

Considering the effects of vaccination, we fitted the effective reproduction number (*R*_*eff*_) globally and for five countries using an MSM model with data from 6 May, 2022, Fig. 5A, which shows that the median *R*_*eff*_ was highest in Germany (3.57 [IQR: 3.36–4.00]), followed by the UK (3.14 [IQR: 2.60–5.37]) and Global (3.11 [IQR: 2.82–5.57]), and Portugal was the lowest (1.05 [IQR:0.98–1.09]), followed by Spain (2.05 [IQR: 1.48–2.34]) and Canada (1.68 [IQR: 1.57–1.90]). Based on the simulation of different steps of *q*, we obtain the distribution of *q* for each country in Fig. 5B. The median of *q* is 2.15% (IQR:1.95–3.84%) for the Global, 2.16% (IQR: 1.80–3.71%) for the UK, 2.46% (IQR: 2.32–2.76%) for Germany, 1.16% (IQR: 1.08–1.31%) for Canada, 0.73% (IQR: 0.68–0.75%) for Portugal and 1.42% (IQR: 1.02–1.62%) for Spain, and the fitted line graph of *q* is detailed in the Additional file 1: Fig. S2.

### Prediction of outbreak trends

**Fig. 6:**
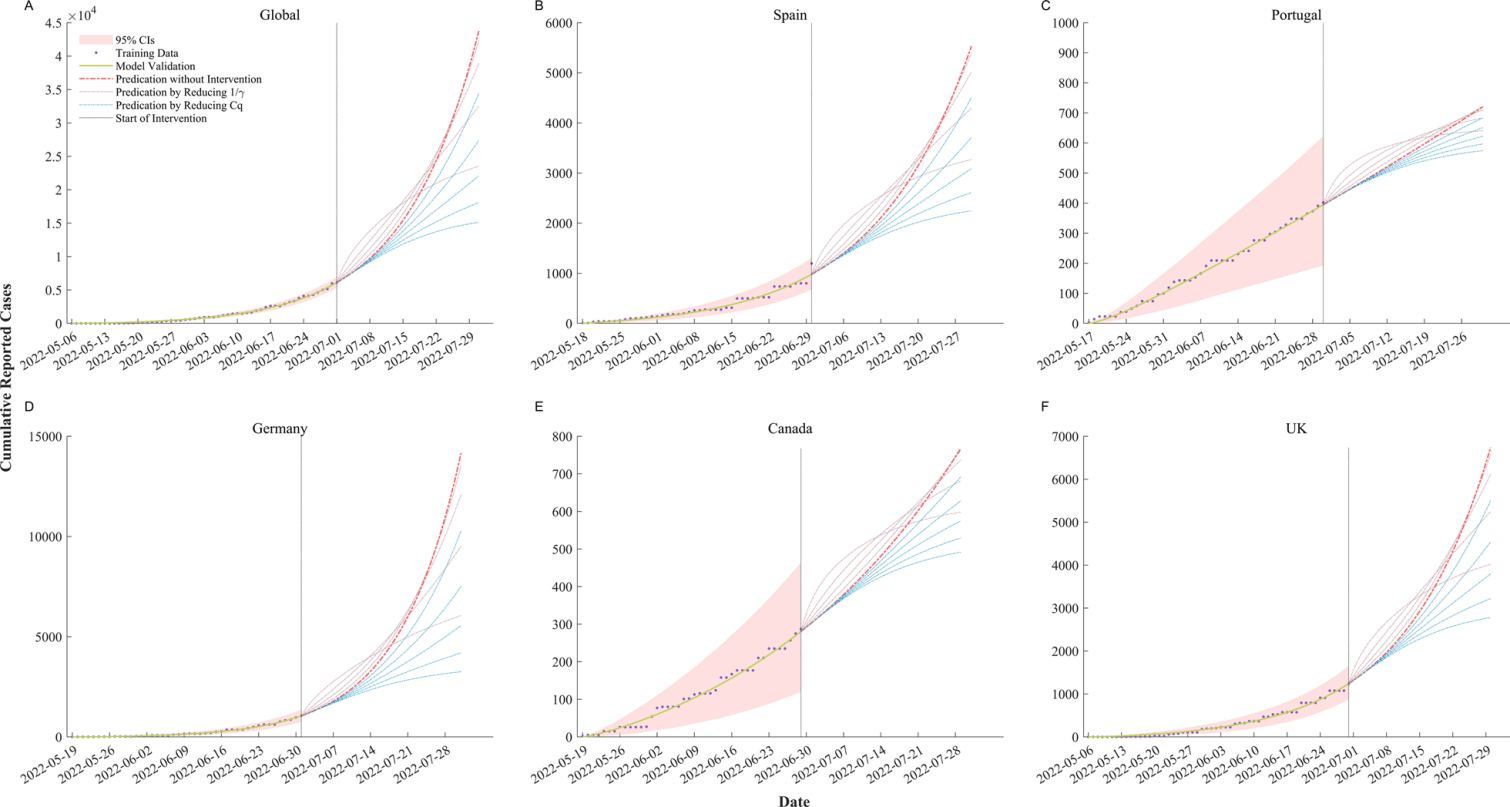
Prediction of outbreak trends of mpox in Global and five selected countries. The purple dots and the green solid line indicate the trends of mpox accumulative cases fitness of our model, and the red shaded area is the 95% confidence interval. The red dash line indicates the trends of mpox accumulative cases when no public health intervention is implemented, while the blue dashed line represents the trend of mpox accumulative cases with different proportions of *Cq* reduction (0.2–1 with a step-size of 20% 20% steps), and the coupled dashed line represents the trend of mpox accumulative cases with different proportions of *1/r* reduction (0.2–0.8 with a step-size of 20%). The prediction period is one month

The model predicts the total number of mpox cases worldwide will reach 43,761 by the end of July when there are not any further interventions taken (Fig. 6). Both data and model prediction shows a near-exponential increase in the total number of mpox cases worldwide. In the current major outbreak areas, such as the UK, the total number of mpox cases will reach 6740 by the end of July if no further public health interventions are implemented. When public health interventions, such as increased social interaction or early case detection that can reduce various degrees of 1*/r* or *Cq* (contact degree and the probability of infection via a single contact), are implemented, there is a trend toward convergence in the total number of mpox cases globally and major outbreak areas (e.g., UK). For instance, when reducing 1*/r* and *Cq* by 60%, the total number of mpox cases worldwide would decrease 25.72% and 49.57% respectively compared to that of no public health intervention, and the total number of mpox cases in major outbreak countries such as the United Kingdom would decrease 22.12% and 43.68% respectively as well.

### Mechanisms of transmission of mpox in the general population

**Fig. 7:**
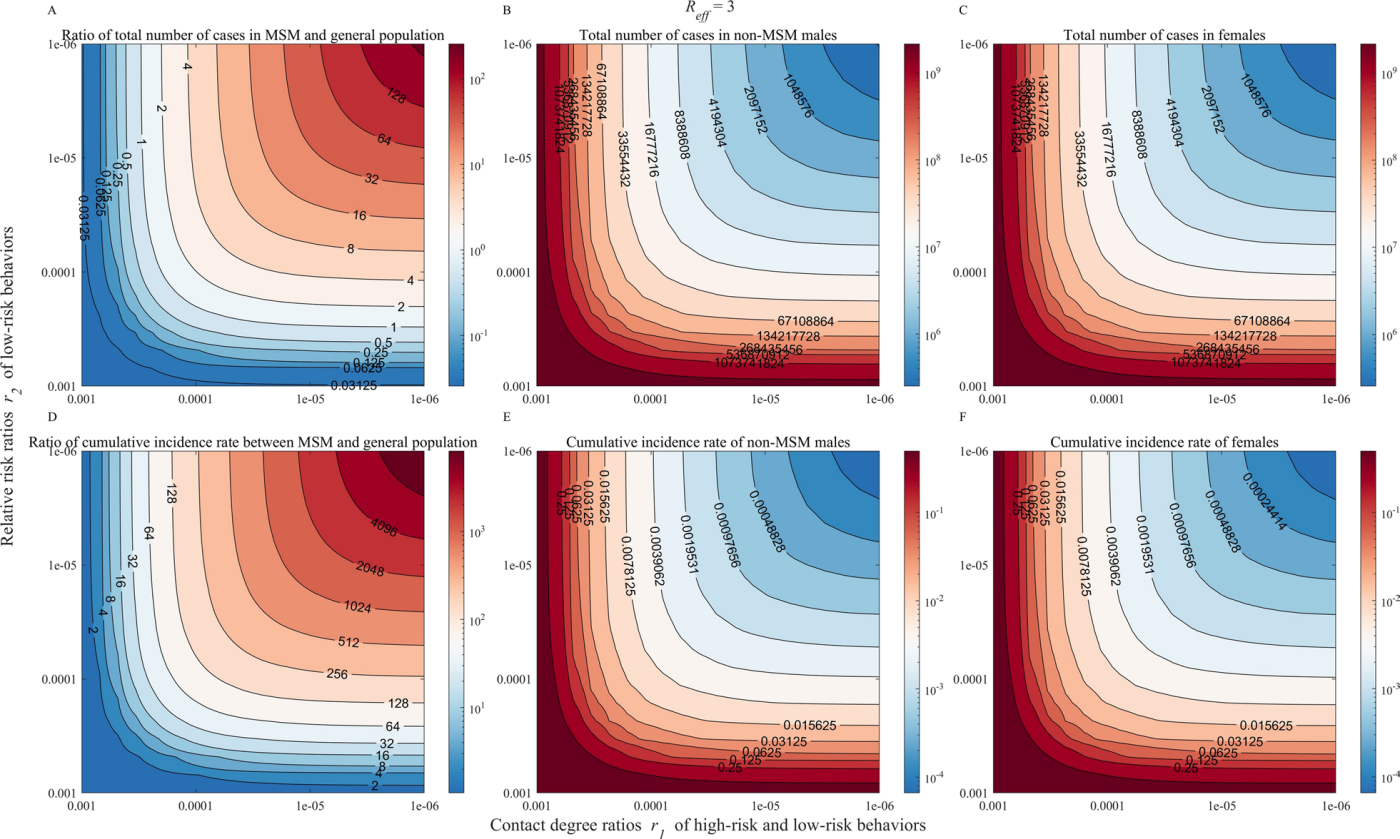
Heatmap of Contact Decomposition. **A** Simulation of the ratio of the total number of cases in MSM and the general population (sum of non-MSM males and females); **B** Simulation of the total number of cases in non-MSM males; **C** Simulation of the total number of cases in females; **D** Simulation of the ratio of cumulative incidence rate between MSM and the general population; **E** Simulation of the cumulative incidence rate of non-MSM males; **F** Simulation of the cumulative incidence rate of females

We quantified risk from MSM to non-MSM by the ratio of cumulative cases/incidence of MSM to cumulative cases/incidence of non-MSM (Fig. 7A, D). When the cumulative number of cases in the general population (the sum of non-MSM males and females) reaches 1 in 32 MSM, the ${r}_{1}$ and ${r}_{2}$ at this point should be less than 10^–5^. Figure 7B and C show that the cumulative number of cases of general populations in contact with high-risk groups (MSM) can reach 4.19 × 10^6^ at both ${r}_{2}$ and ${r}_{1}$ of 10^–5^. Our finding suggests that the cumulative incidence of the general population is much lower (< 2048) than that of MSM when the probability of infection is reduced to less than 1 in 100,000. The cumulative incidence rate in MSM and non-MSM was shown in Table 2. It can be seen from Fig. 7 that the indicators corresponding to the six subplots in the figure are moderately sensitive to the parameters ${r}_{1}$ and ${r}_{2}$. For the most results of *R*_*eff*_ equaled 2–4, see the Additional file 1: Fig. S3-S4.

**Table 2:** The cumulative incidence rate in different risk populations

${r}_{1}$	${r}_{2}$	Cumulative incidence rate in different risk populations		
		MSM	Male	Female
10^–3^	10^–3^	1.26 × 10^–2^	2.56 × 10^–3^	2.53 × 10^–3^
10^–3^	10^–4^	2.65 × 10^–2^	8.66 × 10^–4^	8.57 × 10^–4^
10^–3^	10^–5^	2.66 × 10^–2^	7.18 × 10^–4^	7.11 × 10^–4^
10^–3^	10^–6^	2.66 × 10^–2^	7.05 × 10^–4^	6.97 × 10^–4^
10^–4^	10^–3^	2.65 × 10^–2^	8.45 × 10^–4^	8.36 × 10^–4^
10^–4^	10^–4^	2.62 × 10^–2^	7.01 × 10^–5^	6.94 × 10^–5^
10^–4^	10^–5^	2.61 × 10^–2^	3.64 × 10^–5^	3.61 × 10^–5^
10^–4^	10^–6^	2.61 × 10^–2^	3.33 × 10^–5^	3.29 × 10^–5^
10^–5^	10^–3^	2.66 × 10^–2^	7.01 × 10^–4^	6.93 × 10^–4^
10^–5^	10^–4^	2.61 × 10^–2^	3.64 × 10^–5^	3.60 × 10^–5^
10^–5^	10^–5^	2.61 × 10^–2^	6.27 × 10^–6^	6.20 × 10^–6^
10^–5^	10^–6^	2.60 × 10^–2^	3.43 × 10^–6^	3.39 × 10^–6^
10^–6^	10^–3^	2.66 × 10^–2^	6.87 × 10^–4^	6.80 × 10^–4^
10^–6^	10^–4^	2.61 × 10^–2^	3.32 × 10^–5^	3.29 × 10^–5^
10^–6^	10^–5^	2.60 × 10^–2^	3.42 × 10^–6^	3.39 × 10^–6^
10^–6^	10^–6^	2.60 × 10^–2^	6.19 × 10^–7^	6.13 × 10^–7^

(${r}_{1}$: the contact degree ratios of high-risk and low-risk behavior. ${r}_{2}$: relative risk ratios of low-risk behavior)

### Evaluation of the effectiveness of interventions

**Fig. 8:**
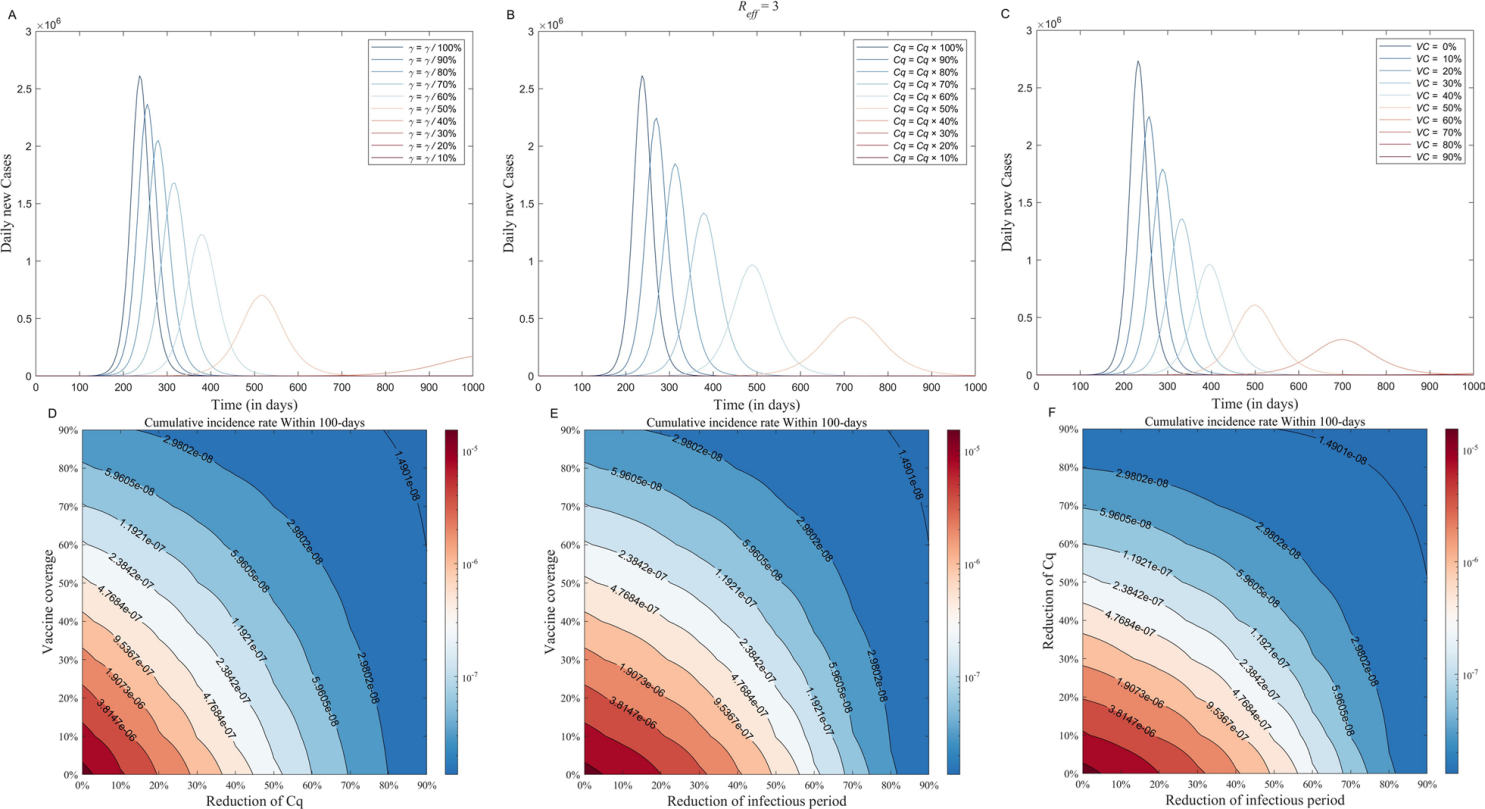
Simulating the effects of single and combined interventions Globally and in five selected countries. The solid lines of **A**–**C** represent the number of daily new cases; the contours of **D**, **E** represent the cumulative incidence rate; **A**: shortened *1/r*; **B**: shortened *Cq*; **C**: increased *VC*; **D**: increased *VC* combined with shortening *Cq*; **E**: increased *VC* combined with shortening *1/r*; **F**: shortened *1/r* combined with shortening *Cq*

The total number of cases among MSM after 1000 days of simulation (*R*_*eff*_ = 3) is 128,574,612; 18,825,355; and 3, respectively, depending on whether the infectious period can be cut by 30%, 60%, and 90% by treatment and early case identification (Fig. 8A). The total number of cases among MSM after 1000 days of simulation is 128,597,550; 189,525; and 3, respectively, depending on whether the *Cq* can be cut by 30%, 60%, and 90% by reducing the frequency of high-risk contact or popularizing protective measures (Fig. 8B). The total number of cases among MSM after 1000 days of simulation is 111,330,610; 56,430,345; and 7, respectively, depending on whether the vaccination coverage can be enhanced by 30%, 60%, and 90% by reducing the frequency of high-risk exposure or popularizing protective measures (Fig. 8C). From Fig. 8A–C we found that all three parameters, 1/*r*, *Cq* and *VC*, are moderately sensitive to the model. The results of the evaluation of the effectiveness of the single intervention were shown in Table 3. The combined intervention results show that (Fig. 8D–F), when we want to control the cumulative incidence of mpox to 1.91 × 10^–6^, we need to increase the vaccine coverage by 34% or reduce the transmission rate factor by 28% or shorten the transmission period by 41%; when we want to control the cumulative incidence to 5.96 × 10^–8^ we need to increase the vaccine coverage by 81% or reduce the transmission rate factor by 70% or shorten the transmission period by 74%. For the most results of *R*_*eff*_ equaled 2–4, see the Additional file 1: Fig. S5-6.

**Table 3:** The evaluation of the effectiveness of the single intervention

Proportion of variation	Total number of cases			Daily incidence peak			Outbreak duration		
	1/*γ*	*Cq*	*VC*	1/*γ*	*Cq*	*VC*	1/*γ*	*Cq*	*VC*
0	147,330,287	147,330,287	150,118,759	237	237	232	501	501	493
0.1	143,350,996	143,341,234	138,262,234	255	270	258	529	559	542
0.2	137,475,836	137,534,913	125,429,890	280	312	288	571	641	603
0.3	128,574,612	128,597,550	111,330,610	318	378	334	634	758	695
0.4	114,498,975	114,515,369	95,564,035	377	489	394	756	968	815
0.5	91,010,395	90,815,781	77,622,136	513	723	500	1011	1380	1007
0.6	18,825,355	189,525	56,430,345	1016	1544	701	1950	2900	1377
0.7	20	20	1,003,433	9	18	1289	10,000	10,000	2454
0.8	5	5	64	5	13	27	10,000	10,000	10,000
0.9	3	3	7	3	11	14	10,000	10,000	10,000

## Discussion

### Epidemiological characteristics

Our finding showed that the recent surge in mpox cases is concentrated in the regions of Europe and America. Overall, an increasing number of mpox cases worldwide are occurring mainly in unvaccinated men, especially among the MSM group. A recent study suggests that current outbreaks in endemic countries may be spread among the gay community through close contact, especially in places where large gatherings or groups meet, such as the Pride event, gay sauna and so on [18–20]. Generally speaking, mpox cases were concentrated in lesbian, gay, bisexual, transgender, queer and plus (LGBTQ +) friendly countries such as the United Kingdom, Spain, Germany, etc. [21]. MSM groups, especially during homosexual gatherings or events, may have higher rates of close contact through anal or genital sexual activity than non-MSM groups [22]. Since smallpox was declared eradicated in 1980 and routine vaccination was subsequently discontinued globally, lack of immunization may have contributed to the recent mpox epidemic, in addition to the increase in high-risk behaviors associated with gatherings [8, 23].

### Transmission of mpox in MSM

In the study, we can see from the *R*_*eff*_ indicators of the globe and in several key countries that the transmissibility of mpox in the MSM in this outbreak is approximately 2.5 (range: 1–4), which is similar to a study using a mathematical model to simulate *R*_0_ of 2.13 and concluded that the DRC has a mpox epidemic potential [15]. However, most studies have calculated the *R*_0_ of the virus to be less than 1 using the African data of mpox [24–27]. This may be explained as a further increase in transmissibility due to clustered activities like Pride events and gay saunas in a high-risk population. In this outbreak, known cases were confirmed to be infected with the milder West African (WA) branch of MPXV, however, according to our findings, this outbreak was able to reach a *R*_*eff*_ of more than 1 despite interventions, which certainly breaks the previous knowledge of the WA branch.

Most of the reported cases in this mpox outbreak were from the MSM population who are at high risk of infection. MPXV may have been introduced into this community by chance due to large gatherings such as gay pride parties [6], and the amplified transmission capacity of mpox can be explained in such a group with a high contact degree scenario and with high-risk contact behaviors. Based on the fitting results, we found that the probability of infection via a single contact with MSM in this outbreak was 2–6%. The probability of infection via a single contact is related to the characteristics of the virus and the susceptibility of the population, but since both factors cannot be artificially altered, specific interventions for this parameter cannot be implemented in real life for policymakers right now. It is reasonable to assume that *q* should be the same for each country, however, lacking detailed contact degrees for MSM populations in each country, we can only use the same set of contact degree data for simulations, which will make *q* vary from country to country.

### Transmission risk in the general population

Most cases reported currently are MSM, but experts claim that mpox is not specified to this population and that anyone who has had close physical contact with an infected person and other high-risk behaviors can result in mpox infection [24], so we simulated the risk from MSM to non-MSM (male and female populations) in the model. ${r}_{1}$ and ${r}_{2}$ has almost no effect on the high-risk population (MSM internal contact is fixed unless the general population affects MSM after transmission), and according to the simulation results, it can be seen that the smaller the *r*_1_/*r*_2_, the larger the ratio of the cumulative number of cases in the high-risk population to the general population, indicating that the general population is safer. We want to reduce the cumulative number of cases in the general population to one percent of the cumulative number of cases in the high-risk population, at least to keep ${r}_{1}$ lower to 10^–5^. Therefore, we need to further control the transmission of mpox in the general population, with a particular focus on reducing the level of contact between the general population and those at high risk for high-risk behaviors.

### Effect of intervention among high-risk population

We predicted the trend of the mpox epidemic in each country based on the fitted *R*_*eff*_ for them, and the results showed that without intervention, the cumulative number of cases will continue to grow rapidly, which will undoubtedly place a certain disease burden on global and individual national health systems. The combined effect of the two interventions showed that community-based prevention and control was the most effective in the first stage of intervention, followed by improved vaccine coverage, and finally early detection/early treatment/isolation. Community-based prevention and control were still the most effective in the second stage when the intervention rate was increased, and early detection/early treatment/isolation was more effective than improved vaccine coverage. Thus, countries, especially those affected by this outbreak, should focus on strengthening community prevention and control, raising people's knowledge and awareness of mpox, promoting safe contact patterns between the general population and confirmed or suspected cases of mpox or high-risk groups, and reducing the frequency of contact to high-risk behaviors.

### Limitation

The mpox surveillance system is not complete yet, and the symptoms of some cases are not typical, so the data of currently reported mpox cases may be underestimated, which may lead us to underestimate the transmissibility of mpox. There are few studies on contact degree by gender or the matrix of contact degree, and relative risk parameters of infection for the occurrence of high-risk behaviors and low-risk behaviors are not available, either. Thus, it is not possible to directly estimate the transmissibility and infection risk of MPXV in non-MSM male and female populations. Finally, the year in which smallpox vaccination was discontinued varies slightly from country to country, but due to a large number of countries worldwide and the difficulty of obtaining data and information, there may be a small impact on our study here.

## Conclusion

The high transmissibility of mpox in MSM required a high level of global concern for this high-risk population. In response to mpox outbreaks, community prevention and control is at the forefront of interventions, as evidenced by reducing contact between high-risk populations and enabling them to take appropriate protective measures, as well as reducing the level of contact between the general population and high-risk populations.

## Supplementary Information

**Additional file 1.** Supplementary material.

## Data Availability

The data we obtained are from the public database (Our world in data, https://ourworldindata.org/monkeypox). The code for the model built in this study has been made openly available for further use at https://github.com/1095912686/Supplementary-Codes-for-Transmission-prevention-and-control-of-human-monkeypox-viral-from-high-risk.

## Abbreviations

WHO: World Health Organization
MSM: Men who have sex with men
IQR: Interquartile range
MPXV: Mpox virus
WA: West African
