# Supplementary material for: Possibility of mpox viral transmission and control from high-risk to the general population: a modeling study
Source: BMC Infect Dis. 2023 Feb 24;23:119. doi: 10.1186/s12879-023-08083-5 (PMC9960212; doi:10.1186/s12879-023-08083-5)
Supplement: Supplementary file 1 — Additional file 1. Supplementary material. [file 12879_2023_8083_MOESM1_ESM.docx]

Supplementary Material

Contents

**S1 Methods1**

S1.1 Data collection2

S1.2 Contact matrices2

S1.3 Natural history and various human behavior6

S1.4 Vaccination effectiveness and coverage8

S1.5 Demographic data9

S1.6 Sensitivity analysis9

S1.7 Transmissibility estimation10

S1.8 Forward Piecewise Fitting10

S1.9 The evaluation of the effectiveness of the single intervention.12

S1.10 Table S112

**S2 Additional results13**

S2.1 Figure S113

S2.2 Figure S214

S2.3 Figure S315

S2.4 Figure S416

S2.5 Figure S517

S2.6 Figure S618

**S3 Reference for the Supplementary Appendix19**

# S1. Methods

**S1.1** **Data collection**

The database contains case information for all countries worldwide where mpox cases were reported between May 6 and July 1, 2022, with the majority of confirmed mpox cases coming from 33 countries in the European region (United Kingdoms, Spain, Portugal, etc.) [1], other regions of mpox confirmed cases include: 10 countries in America region (United States, Mexico, Canada, Argentina, etc.), 3 countries in Eastern Mediterranean region (United Arab Emirates, Israel, Morocco), 4 countries in Western Pacific region (Australia, China, Republic of Korea, etc.), and 3 countries in Africa region (Ghana, Benin, South Africa). Case information includes case ID, status, country, city, age, sex, date of onset, date of diagnosis, symptoms, travel history, and location of travel, as well as the source of data for each country. Since data from the UK showed a history of foreign travel in 22% of cases [2], this study multiplied the UK case data by a rate of 0.78 to account for the effect of imported cases when estimating transmissibility [3]. However, the impact of imported cases on other countries was not considered for the time being, because the rate of imported cases was not reported by them.

# S1.2 Contact Matrices

**Definition**

**Definition:**(Contact Matrix)
The contact matrix $C$ is a square matrix with its $ij$-th entry $c_{ij}$ denotes the average number of daily contacts in group $j$ produced by an individual in group $i$.

**Basic Properties of the Contact Matrix**

We consider a bipartite graph of group $i$ and $j$, by omitting their inner edges and edges connecting other groups. In group $i$, there are $N_{i}$ individuals denoted by vertices $V_{1},V_{2},\cdots,V_{N_{i}}$; and for group $j$, the $N_{j}$ individuals are denoted by vertices $W_{1},W_{2},\cdots,W_{N_{j}}$. Each contact data pair is denoted by an edge connect group $i$ and group $j$.
S1.2 - Figure 1 reveals the relation between $c_{ij}$ and $c_{ji}$.

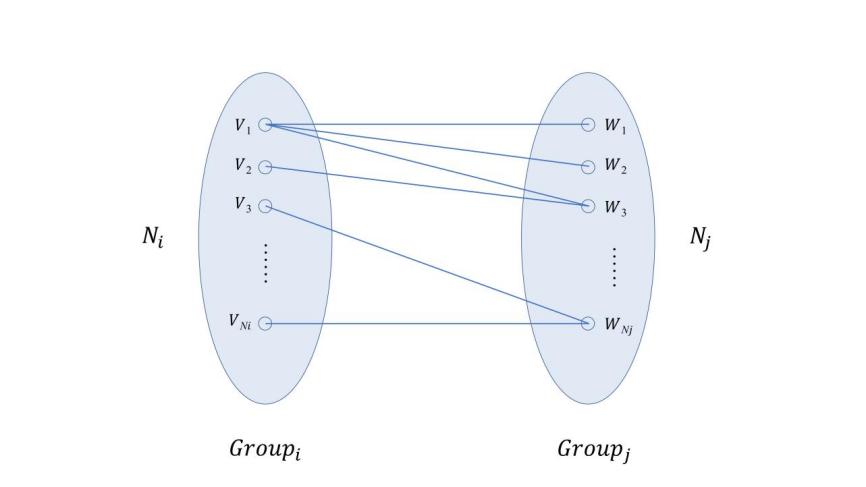


S1.2 - Figure 1 Bipartite Graph Model

In graph theory, the degree of a vertex is defined as the number of edges joined to that vertex.
By definition of contact matrix, $c_{ij}$ is the average degree of vertices in group $i$ (in this bipartite graph), and thus by multiplying $N_{i}$, one obtains the number of edges. Now we have an equation: number of edges computed from left (group i) equals to number of edges computed from right (group j), that is:

$$\begin{matrix} c_{ij}N_{i}=c_{ji}N_{j} \end{matrix}$$

**Contact Matrices with Assumptions on Mixing Patterns**

Summing contact matrix $C$ by rows, and denote the result column vector as $\vec{c}$. The $\vec{c}$ reflects the contact frequency of individuals in each group (with its $i$-th component, $c_{i}$, represent the average daily number of contacts produced by one individual in group $i$).
A normalized probability matrix $P$ with $\sum_{j} p_{ij}=1, \forall i$ is introduced to describe the distribution of contacts made in $c_{i}$ among groups. That is, the contact matrix is formularized as:

$$\begin{matrix} C=P\odot\vec{c}, \end{matrix}$$

where $\odot$ is the entry-wise product.
Three mixing patterns: restricted, proportional and preferred mixing that gives different structures of $P$ are discussed at <https://www.sciencedirect.com/science/article/abs/pii/0025556488900314>.

**Restricted Mixing**

The restricted mixing assumes all contacts happen only inside each group, that is,

$$\begin{matrix} P_{1}=\left( \begin{matrix} 1 & 0 & 0 \\ 0 & 1 & 0 \\ 0 & 0 & 1 \end{matrix} \right) \end{matrix}$$

**Proportional Mixing**

The proportional mixing assumes the fraction of the contacts of group i with group j is equal to the fraction of total contacts made by the population that are due to group j, that is,

$$\begin{matrix} p_{ij}=\frac{c_{j}N_{j}}{\sum c_{j}N_{j}}, \end{matrix}$$

and

$$\begin{matrix} P_{2}=\left( \begin{matrix} 1 \\ 1 \\ 1 \end{matrix} \right)\frac{\left( \vec{c}\odot\vec{N} \right)^{T}}{\|\vec{c}\odot\vec{N}{\|}_{1}} \end{matrix}$$

where $\vec{N}$ is the vector of population size in each group.

**Preferred Mixing**

The preferred mixing is a linear combination of restricted and proportional mixing. A group-relevant ratio vector $\vec{r}$ is introduced, with each entry lying between 0 and 1 (representing the fraction of Restricted Mixing and Proportional Mixing in corresponding groups). Then the group-wise linear combination gives:

$$\begin{matrix} P_{3}=\vec{r}\odot P_{1}+\left( 1-\vec{r} \right)\odot P_{2} \end{matrix}$$

**Profiles**

**Values**

Group Names:

$$\begin{matrix} groupNames=\left( \begin{matrix} \text{MSM} \\ \text{Male not MSM} \\ \text{Female} \end{matrix} \right). \end{matrix}$$

Standard Contact vector $\vec{c}$:

$$\begin{matrix} \vec{c}=\left( \begin{matrix} 13.51 \\ 13.51 \\ 13.39 \end{matrix} \right). \end{matrix}$$

The parameters of the contact matrix were set with reference to a European study of Social Contacts and Mixing Patterns in eight countries[4], where the male was contacted by 13.51 people per day and female by 13.39 people per day. Contact was defined as physical contact with acts such as kissing or shaking hands, or non-physical contact with another person with more than three two-way conversations.

Population vector:

$$\begin{matrix} \vec{N}=\left( \begin{matrix} 4030711645*0.04 \\ 4030711645*0.96 \\ 3957932033*1.00 \end{matrix} \right) \end{matrix}$$

Assume that the proportional mixing is dominant in the population, one obtains the contact matrix:

$$\begin{matrix} C & =\vec{c}\odot P_{2} \\ & =\left( \begin{matrix} 0.2739 & 6.573 & 6.663 \\ 0.2739 & 6.573 & 6.663 \\ 0.2714 & 6.514 & 6.604 \end{matrix} \right) \end{matrix}$$

**S1.3** **Natural history and various human behavior**

While mpox virus was first transmitted from animals (African rodents and non-human primates) to humans, the recent surge in risky behaviors in MSM has led to a rapid increase in human-to-human transmission cases. In an infectious procedure, a susceptible person would become infected after contacting the areas that have been contaminated by cases. Before experiencing symptoms, most exposed individuals would go through an incubation period. The National Health Service (NHS) publishes on its website that it takes 5-21 days for symptoms to appear in mpox infections [5], and the US CDC website shows that the incubation period for mpox is usually 7-14 days, but ranges from 5-21 days [6]. The Netherlands investigated 18 confirmed cases as of 31 May 2022, which showed a mean incubation period of 8.5 days (range: 4.2-17.3) [7]. The 2022 Italian study analyzed 255 PCR-confirmed cases of mpox indicating mean incubation period of 9.1 (95% CI 6.5-10.9) days [8]. A cross-sectional study involving 18 sites in 15 countries showed a median incubation period of 8 (IQR: 5-11) days for mpox [9]. We have taken the median incubation period of 8.5 based on these latest research articles in 2022 [7-9]. Our model, therefore, sets *ω* to 1/8.5. Most mpox virus infections are recoverable. A study reported that the duration of symptoms of mpox usually lasts 2-4 weeks and recovery is possible without treatment [10]. Most instances of transmission occur well before symptoms have subsided. They could have been confined at this stage, rendering them non-contagious. Based on data collected in the UK for mpox cases in April-May 2022 indicating that the median number of days from onset to reporting was 11 days (interquartile range (IQR): 6-14 days) [11], we set the *γ* to 1/11 in the model (Table S1).

In this model, we set *β* = *C* × *q*, where *q* refers to the probability of infection via a single contact and *C* refers to the contact matrix, whose -th entry represents the daily average number of contacts in group that one individual in group made. As the most important risk factors for mpox infection, close physical contact and large droplet transmission through the respiratory tract, are specifically considered by dividing the contact matrix *C* into high-risk behavioral contacts and low-risk behavioral contacts.

High-risk behavioral contact was defined as sexual contact with multiple or different sexual partners in the previous 3 weeks before symptom onset. The contact degree for high-risk behavior is denoted as and the corresponding probability of infection for a single contact is denoted as. The mean number of partners, population size, and daily probability of having a one-time sexual contact are listed in detail in a US study of sexual networks in the transmission of mpox virus among MSM [12]. We focused on the strata with one-time partners and weighted the population proportions according to different sexual activity stratums and set them to 0.0234.

Low-risk behavioral contact was defined as all other behaviors other than the high-risk contact behaviors defined above (such as sexual contact regular sexual partners, contact with objects contaminated with mpox virus and air, droplet contact transmission) were defined as low-risk contact behaviors. The contact degree for the low-risk behavioral contact being and the probability of infection via a single contact of transmission being.

**S1.4** **Vaccination effectiveness and coverage**

Three studies, based on data of mpox cases in Zaire from 1980-1984, compared the difference in the incidence of mpox infection between unvaccinated and vaccinated contacts and concluded that smallpox vaccination gave approximately 85% protection against mpox infection [13-15]. The vaccine effectiveness of smallpox in preventing mpox infection is also stated as 85% on the WHO website in the mpox vaccination section, so the *VE* was also set to 85% in our study [16].

Due to the eradication progress for smallpox before 1980, mass vaccination was implemented globally and achieved 80% smallpox vaccine coverage (*VC*) on average in all countries. The vaccination has stopped since smallpox has been eliminated in 1980, leaving people born after 1990 susceptible to mpox. Currently, the mpox vaccine coverage rate of 80% is among the population aged 42 years and older. According to the composition of the global population age group, 23.1% of the total global population is aged 40-59 years; 36.81% of the population is aged 40 years or older [17], so we estimate that 34.5% of the population is aged 42 years or older.

**S1.5** **Demographic data**

All values about the population are obtained from global data. According to Statistics Times, a professional statistics agency, the size of the global male population is 3,970,238,390 and the male population is 3,904,727,342 [18]. Since the MSM population takes 4% of the total male population [18], the size of the global MSM population was set to be 1.588 × 10^8^, the global non-MSM male population was set to be 3.811×10^9^, and the total number of the female population was set as 3.905 × 10^9^ for this study.

**S1.6** **Sensitivity analysis**

Since the SEIR models with multi-group groups are widely used by many studies, the sensitivity of other model parameters could be found in those references [19, 20]. In our model, the vector of VE, is multiplied on the group-wise contact matrix; which makes the sensitivity analysis analogous to those for the contact matrix.

**S1.7 Transmissibility estimation**

The transmissibility of the MPXV was quantified using the basic reproduction number (*R_0_*) and effective reproduction number (*R_eff_*) [21]. When intervention measures are taken, we use *R_eff_* to estimate the transmissibility of MPXV. *R_eff_* refers to the expected number of secondary infections that result from introducing a single infected individual into a wholly susceptible population when public health interventions are implemented [22]. When *R_eff_* > 1, the disease will spread and cause an epidemic; while *R_eff_* < 1 indicates the epidemic will soon die out; and *R_eff_* = 1 means the disease will neither cause an epidemic nor stop. The formula for *R_eff_* in the SEIR model is as follows:

$$\begin{matrix} Reff & =\lambda_{\max}(\beta\odot N)/r \\ & =\frac{q\cdot\lambda_{\max}(C\odot(1-VE))}{\gamma} \end{matrix}$$

Where $\lambda_{\max}()$ represent the maximum real part of the eigenvalue of the matrix.

**S1.8 Forward Piecewise Fitting**

We calibrate the probability of infection via a single contact using reported incidence data. To handle the uncertainty of data noise, we adopted a forward-piecewise-fitting method, which fits one for each data segment, and summarizes the distribution of those.


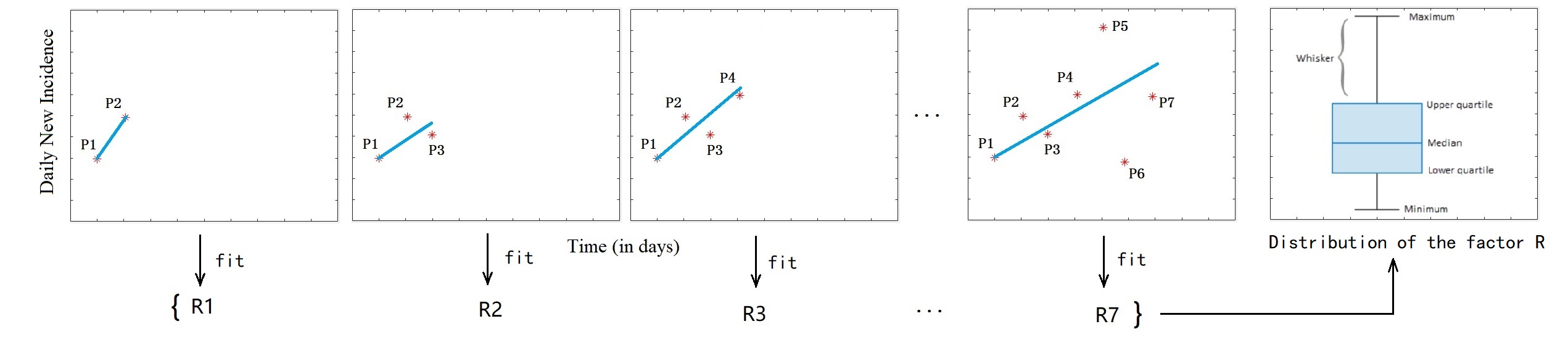


For computing without any intervention, we shall use the incidence data segment as early as possible in the stage of transmission, however, this conflicts to a relatively long segment demanded a stable fitting (stable to noise in daily new incidence). The shortest and longest length of segments is crucial parameters for summarizing the distribution of factors. Although in practice, one can always fit all possible segments and then select these parameters, we choose the first 20 observations of daily new incidence as the shortest segment, and the entire data set (until 2022/06/17) for the longest segment.

**S1.9 Intervention simulation**

We assumed that the infected population could reduce the infectious period through early detection/ early treatment/ isolation, the susceptible population could reduce the risk of infection by reducing the frequency of high-risk contact and popularizing protection measures, and the susceptible population could acquire immunity after vaccination. We simulated the intervention effects by varying the parameters 1/*γ*, *Cq* and *VC*.

| Parameter | Description | Unit | Value | Range | Source |
| --- | --- | --- | --- | --- | --- |
| *β* | The transmission rate | day^−1^ | - | ≥0 | Curve fitting |
| *C* | Contact degree | (Person × day) ^−1^ | - | ≥0 | Reference [4, 23] |
| *q* | Probability of infection via a single contact | 1 | - | ≥0 | Curve fitting |
| *1/ω* | Incubation period | day^−1^ | 8.5 | 5-21 | Reference [7, 24-26] |
| *1/γ* | Infectious period | day^−1^ | 11 | 6-14 | Reference [11] |
| *VE* | Vaccine efficacy of smallpox | 1 | 0.85 | ≥0 | Reference  [13-15, 26] |
| *VC* | Vaccine coverage of smallpox | 1 | 0.8 | ≥0 | Reference [27] |
| *N* | The number of each group | 1 | - | ≥0 | Reference [28, 29] |

**Table S1.** **Parameter values used in the model.**

**S2. Additional results**


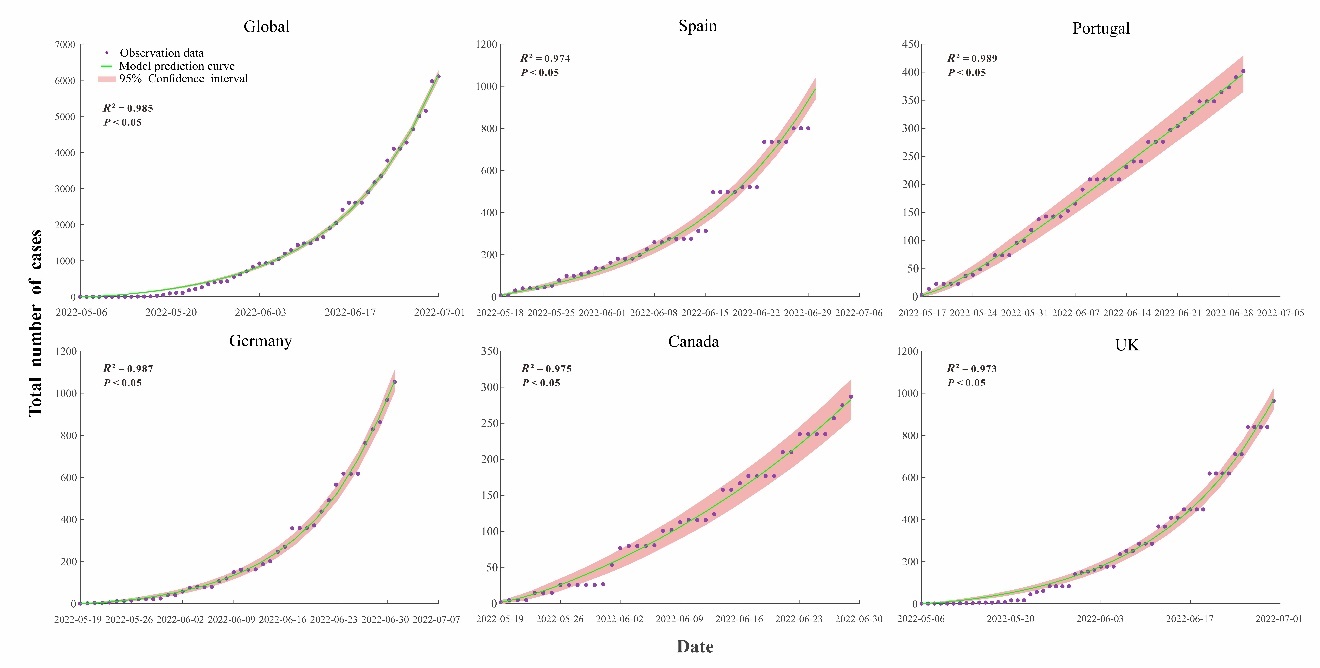


**Figure S1.** Curve fitting of the high-risk (MSM) SEIR model to the cumulative data in Global and five selected countries.


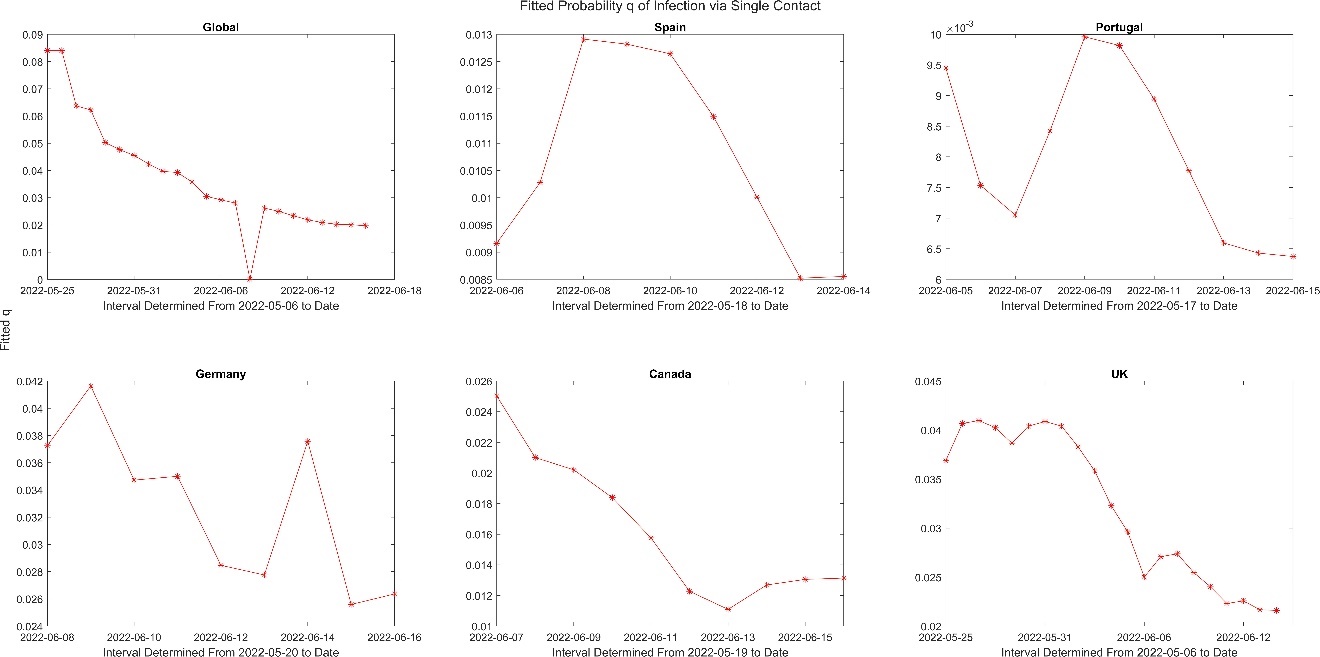


**Figure S2.** Fitting results of the probability of infection via a single contact (*q*).

.


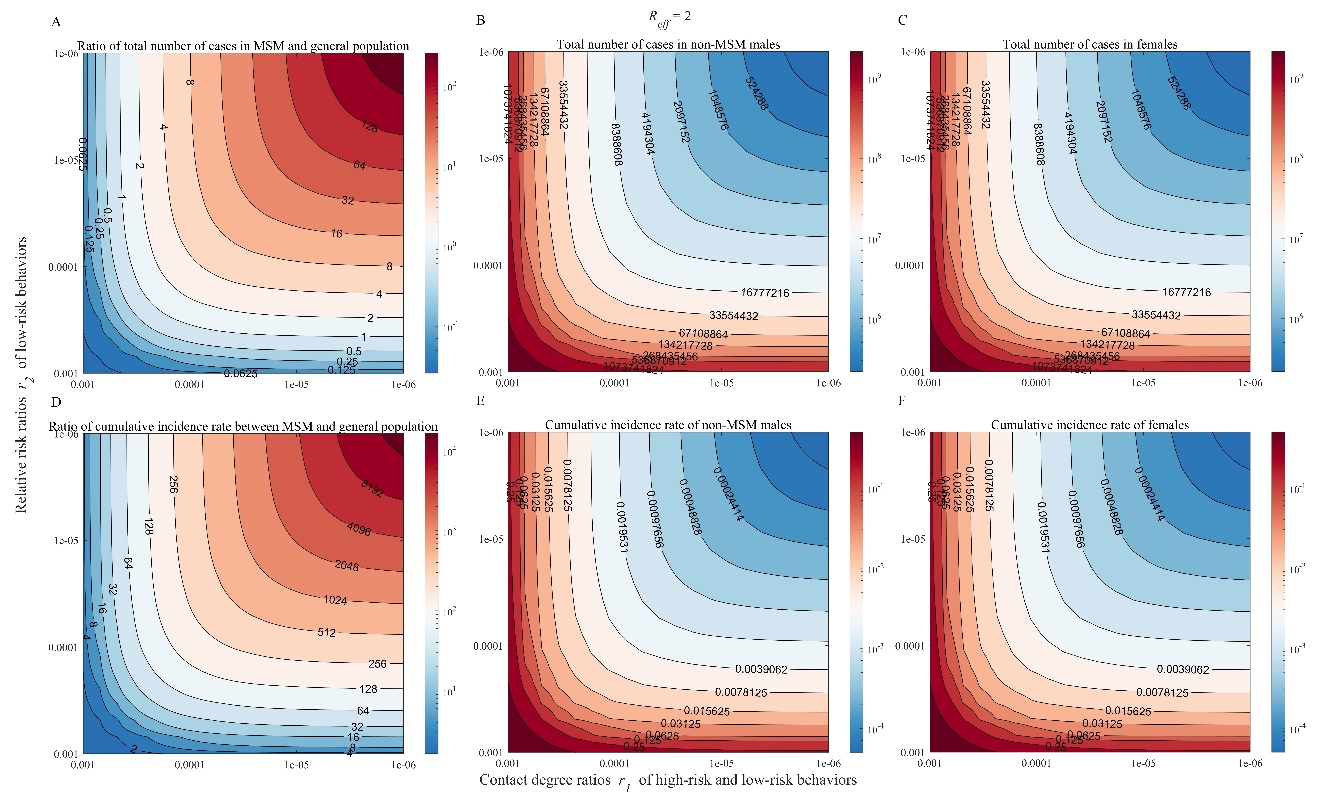


**Figure S3.** Heatmap of Contact Decomposition (*R_eff_* = 2). A: Simulation of the ratio of the total number of cases in MSM and the general population (sum of non-MSM males and females); B: Simulation of the total number of cases in non-MSM males; C: Simulation of the total number of cases in females; D: Simulation of the ratio of cumulative incidence rate between MSM and the general population; E: Simulation of the cumulative incidence rate of non-MSM males; F: Simulation of the cumulative incidence rate of females.


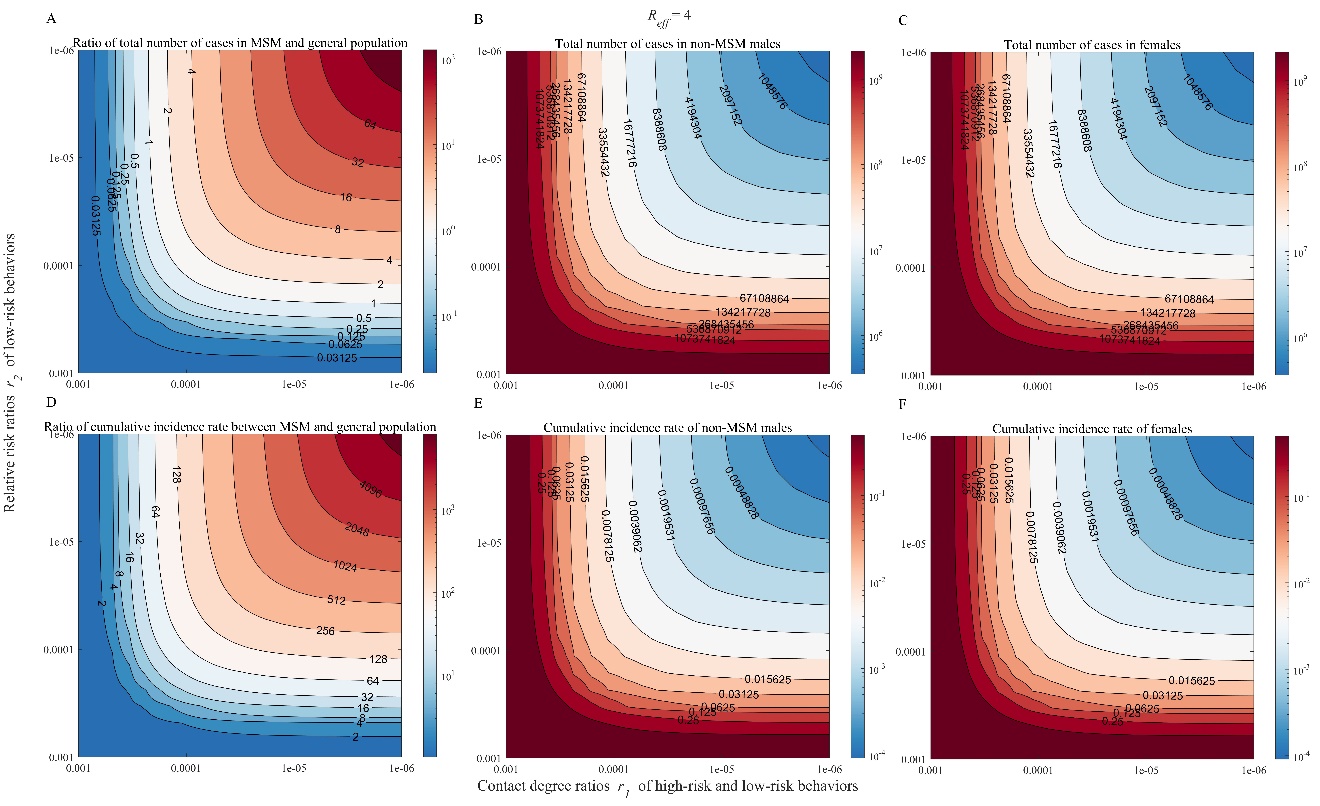


**Figure S4.** Heatmap of Contact Decomposition (*R_eff_* = 4). A: Simulation of the ratio of the total number of cases in MSM and the general population (sum of non-MSM males and females); B: Simulation of the total number of cases in non-MSM males; C: Simulation of the total number of cases in females; D: Simulation of the ratio of cumulative incidence rate between MSM and the general population; E: Simulation of the cumulative incidence rate of non-MSM males; F: Simulation of the cumulative incidence rate of females.

**
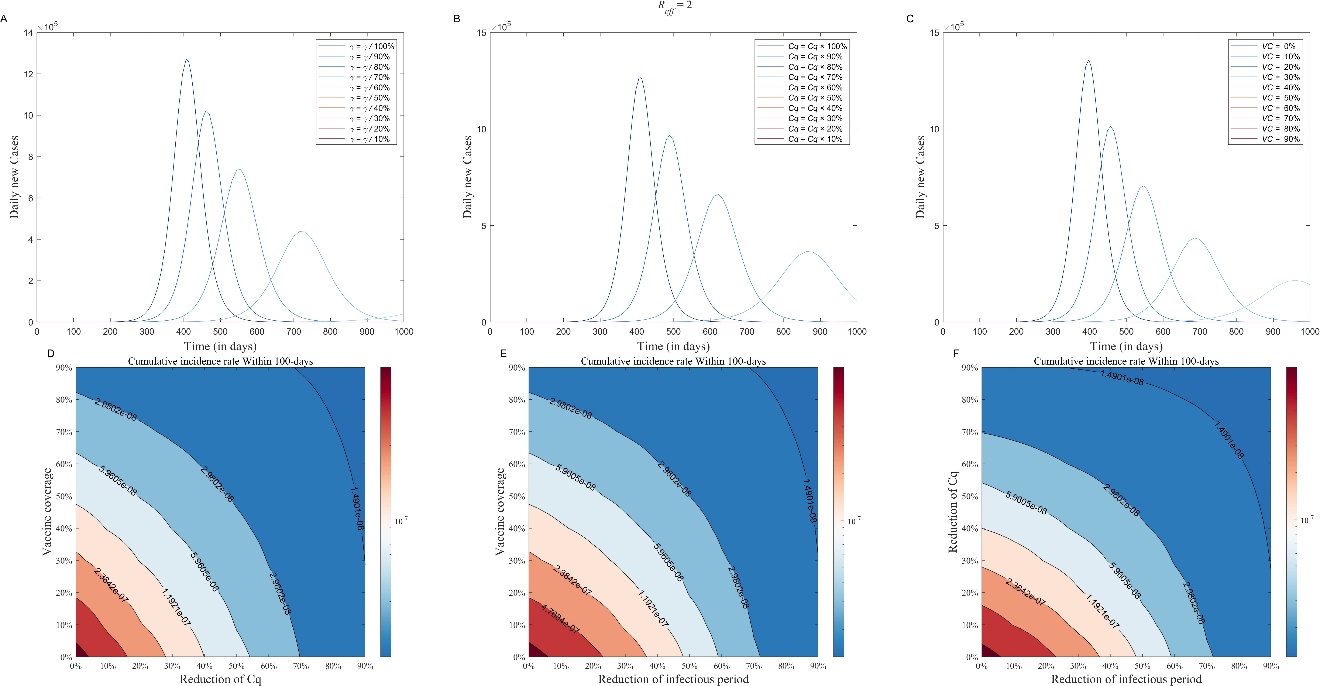
**

**Figure S5.** Simulating the effects of single and combined interventions Global and five selected countries (*R_eff_* = 2). The solid lines of A-C represent the number of daily new cases; the contours of D-E represent the cumulative incidence rate; A: shortened *1*/*r*; B: shortened *Cq*; C: increased *VC*; D: increased *VC* combined with shortening *Cq*; E: increased *VC* combined with shortening *1*/*r*; F: shortened *1*/*r* combined with shortening *Cq*.


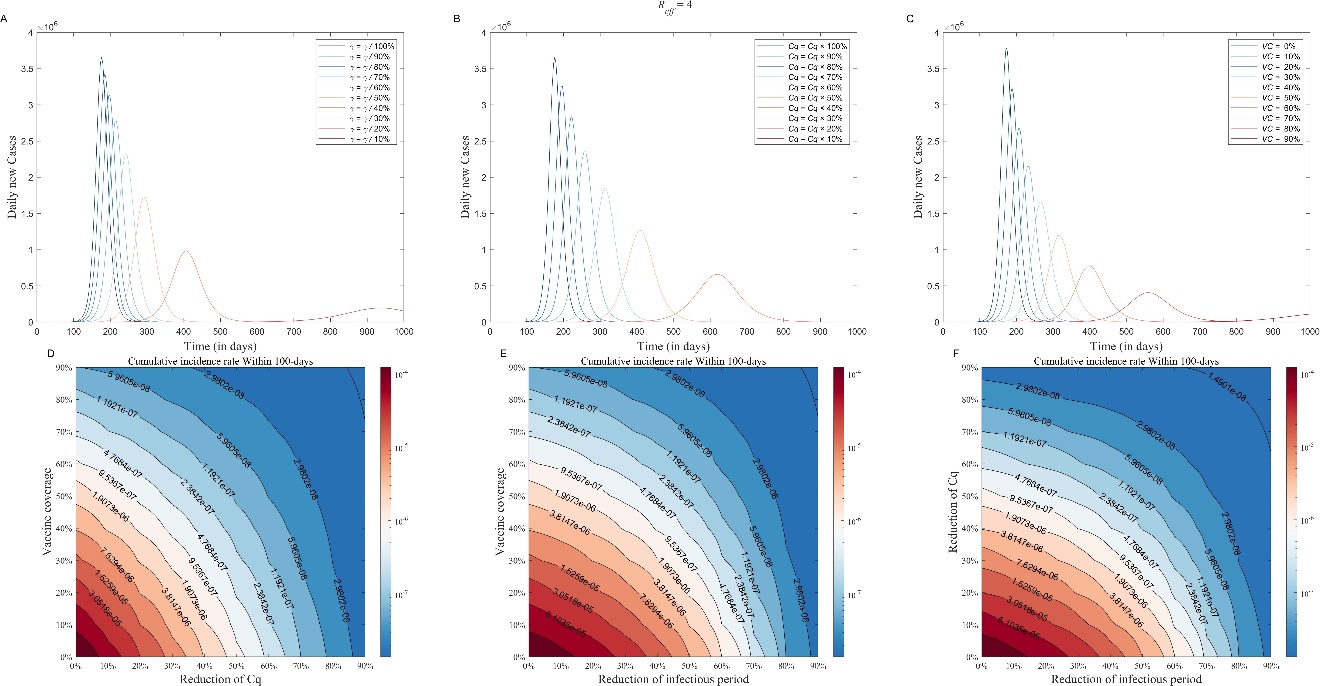


**Figure S6.** Simulating the effects of single and combined interventions Global and five selected countries (*R_eff_* = 4). The solid lines of A-C represent the number of daily new cases; the contours of D-E represent the cumulative incidence rate; A: shortened *1*/*r*; B: shortened *Cq*; C: increased *VC*; D: increased *VC* combined with shortening *Cq*; E: increased *VC* combined with shortening *1*/*r*; F: shortened *1*/*r* combined with shortening *Cq*.

**S3. Reference**

1. Countries [https://www.who.int/countries/]

2. Investigation into monkeypox outbreak in England: technical briefing 1 [https://www.gov.uk/government/publications/monkeypox-outbreak-technical-briefings/investigation-into-monkeypox-outbreak-in-england-technical-briefing-1]

3. Multi-country monkeypox outbreak in non-endemic countries [https://www.who.int/emergencies/disease-outbreak-news/item/2022-DON385]

4. Mossong J, Hens N, Jit M, Beutels P, Auranen K, Mikolajczyk R, Massari M, Salmaso S, Tomba GS, Wallinga J et al: Social contacts and mixing patterns relevant to the spread of infectious diseases. PLoS Med 2008, 5(3):e74.

5. Monkeypox [https://www.nhs.uk/conditions/monkeypox/]

6. Monkeypox: Signs and Symptoms [https://www.cdc.gov/poxvirus/monkeypox/symptoms.html]

7. Miura F, van Ewijk CE, Backer JA, Xiridou M, Franz E, Op de Coul E, Brandwagt D, van Cleef B, van Rijckevorsel G, Swaan C et al: Estimated incubation period for monkeypox cases confirmed in the Netherlands, May 2022. Euro Surveill 2022, 27(24).

8. Guzzetta G, Mammone A, Ferraro F, Caraglia A, Rapiti A, Marziano V, Poletti P, Cereda D, Vairo F, Mattei G et al: Early Estimates of Monkeypox Incubation Period, Generation Time, and Reproduction Number, Italy, May-June 2022. Emerg Infect Dis 2022, 28(10):2078-2081.

9. Angelo KM, Smith T, Camprubí-Ferrer D, Balerdi-Sarasola L, Díaz Menéndez M, Servera-Negre G, Barkati S, Duvignaud A, Huber KLB, Chakravarti A et al: Epidemiological and clinical characteristics of patients with monkeypox in the GeoSentinel Network: a cross-sectional study. Lancet Infect Dis 2022.

10. Damon IK: Status of human monkeypox: clinical disease, epidemiology and research. Vaccine 2011, 29 Suppl 4:D54-59.

11. Vivancos R, Anderson C, Blomquist P, Balasegaram S, Bell A, Bishop L, Brown CS, Chow Y, Edeghere O, Florence I et al: Community transmission of monkeypox in the United Kingdom, April to May 2022. Euro Surveill 2022, 27(22).

12. Spicknall IH, Pollock ED, Clay PA, Oster AM, Charniga K, Masters N, Nakazawa YJ, Rainisch G, Gundlapalli AV, Gift TL: Modeling the Impact of Sexual Networks in the Transmission of Monkeypox virus Among Gay, Bisexual, and Other Men Who Have Sex with Men - United States, 2022. MMWR Morb Mortal Wkly Rep 2022, 71(35):1131-1135.

13. Fine PE, Jezek Z, Grab B, Dixon H: The transmission potential of monkeypox virus in human populations. Int J Epidemiol 1988, 17(3):643-650.

14. Jezek Z, Marennikova SS, Mutumbo M, Nakano JH, Paluku KM, Szczeniowski M: Human monkeypox: a study of 2,510 contacts of 214 patients. J Infect Dis 1986, 154(4):551-555.

15. Jezek Z, Grab B, Paluku KM, Szczeniowski MV: Human monkeypox: disease pattern, incidence and attack rates in a rural area of northern Zaire. Trop Geogr Med 1988, 40(2):73-83.

16. Detail/Monkeypox [https://www.who.int/news-room/fact-sheets/detail/monkeypox]

17. Visualizing the World’s Population by Age Group [https://www.visualcapitalist.com/the-worlds-population-2020-by-age/]

18. Gender ratio in the World [https://statisticstimes.com/demographics/world-sex-ratio.php]

19. Zhao ZY, Zhu YZ, Xu JW, Hu SX, Hu QQ, Lei Z, Rui J, Liu XC, Wang Y, Yang M et al: A five-compartment model of age-specific transmissibility of SARS-CoV-2. Infect Dis Poverty 2020, 9(1):117.

20. Zhao Z, Chen Q, Wang Y, Chu M, Hu Q, Hannah MN, Rui J, Liu X, Yu Y, Zhao F et al: Relative transmissibility of shigellosis among different age groups: A modeling study in Hubei Province, China. PLoS Negl Trop Dis 2021, 15(6):e0009501.

21. Shah NH, Mittal M: Introduction to Compartmental Models in Epidemiology. In: Mathematical Analysis for Transmission of COVID-19: 2021// 2021; Singapore: Springer Singapore; 2021: 1-20.

22. Lim JS, Cho SI, Ryu S, Pak SI: Interpretation of the Basic and Effective Reproduction Number. J Prev Med Public Health 2020, 53(6):405-408.

23. Wall KM, Stephenson R, Sullivan PS: Frequency of sexual activity with most recent male partner among young, Internet-using men who have sex with men in the United States. J Homosex 2013, 60(10):1520-1538.

24. Monkeypox [https://www.nhs.uk/conditions/monkeypox/]

25. Monkeypox: Signs and Symptoms [https://www.cdc.gov/poxvirus/monkeypox/symptoms.html]

26. Detail/Monkeypox [https://www.who.int/news-room/fact-sheets/detail/monkeypox]

27. Visualizing the World’s Population by Age Group [https://www.visualcapitalist.com/the-worlds-population-2020-by-age/]

28. Gender ratio in the World [https://statisticstimes.com/demographics/world-sex-ratio.php]

29. Demographics of sexual orientation [https://en.wikipedia.org/wiki/Demographics_of_sexual_orientation]

# 
